# Adaptive learning in a compartmental model of visual cortex—how feedback enables stable category learning and refinement

**DOI:** 10.3389/fpsyg.2014.01287

**Published:** 2014-12-05

**Authors:** Georg Layher, Fabian Schrodt, Martin V. Butz, Heiko Neumann

**Affiliations:** ^1^Institute of Neural Information Processing, Ulm UniversityUlm, Germany; ^2^Department of Computer Science, University of TübingenTübingen, Germany

**Keywords:** neural model, category learning, subcategory learning, unsupervised learning, feedforward and feedback processing

## Abstract

The categorization of real world objects is often reflected in the similarity of their visual appearances. Such categories of objects do not necessarily form disjunct sets of objects, neither semantically nor visually. The relationship between categories can often be described in terms of a hierarchical structure. For instance, tigers and leopards build two separate mammalian categories, both of which are subcategories of the category Felidae. In the last decades, the unsupervised learning of categories of visual input stimuli has been addressed by numerous approaches in machine learning as well as in computational neuroscience. However, the question of what kind of mechanisms might be involved in the process of subcategory learning, or category refinement, remains a topic of active investigation. We propose a recurrent computational network architecture for the unsupervised learning of categorial and subcategorial visual input representations. During learning, the connection strengths of bottom-up weights from input to higher-level category representations are adapted according to the input activity distribution. In a similar manner, top-down weights learn to encode the characteristics of a specific stimulus category. Feedforward and feedback learning in combination realize an associative memory mechanism, enabling the selective top-down propagation of a category's feedback weight distribution. We suggest that the difference between the expected input encoded in the projective field of a category node and the current input pattern controls the amplification of feedforward-driven representations. Large enough differences trigger the recruitment of new representational resources and the establishment of additional (sub-) category representations. We demonstrate the temporal evolution of such learning and show how the proposed combination of an associative memory with a modulatory feedback integration successfully establishes category and subcategory representations.

## 1. Introduction

Stimuli presented in isolation cause cortical responses by feeding a representation defined by the feature arrangement that is contained in the current scene. The strength of the response depends on its contrast but is influenced by the local context in which it is embedded. Such (local) context information is integrated and thus made available at a neural site via lateral intra-cortical interactions, preferentially through long-range associative interactions in the superficial layers of cortex (Self et al., 2012). Larger context is integrated through the hierarchical processing of inputs over several stages of the cortical hierarchy where feature specificity of the neurons becomes more and more specific, integrating over an increasingly more widespread space-feature domain (Markov and Kennedy, 2013). At earlier stages, the result of such feature integration is made available via top-down feedback to merge feature representations of higher levels with spatially more localized responses from initial filtering. Such convergence of feedforward and feedback streams of activation has recently been demonstrated to occur at the level of individual cortical columns (Mountcastle, 1997; Larkum, 2013).

Feedback signals tend to modulate the responses of activations at the earlier representations of raw feature presence (Larkum et al., 2004; Self et al., 2013). Modulating interactions are a common principle of neuronal interaction, which have been observed at different levels of cortical processing, subserving different cognitive computational functions, such as attention, figure-ground segregation, or grouping (Roelfsema et al., 2007; Poort et al., 2012). However, the precise functional role of feedback signals along downstream pathways is largely unclear and a topic of intense research investigation. Specific theoretical frameworks have been proposed that receive support by recent experimental investigations (Markov and Kennedy, 2013). One such theoretical framework proposes that feedforward sensory activations are amplified by matching feedback such that those cells yield enhanced activations in a competition of cells, that have received a competitive advantage via modulating feedback (biased competition; Girard and Bullier, 1989; Desimone, 1998). Another framework considers the role of feedback as a predictive signal in which a template is activated that predicts the expected input given the evidence derived from current bottom-up input signals. The interaction of feedforward and feedback signals reduces the residual discrepancy between the different signal streams (Ullman, 1995; Rao and Ballard, 1999; Bastos et al., 2012). Overall, the literal difference between these model frameworks lies in the different roles feedback exerts on the bottom-up driven representations, although under certain conditions the two frameworks yield two variants of the same generic principles (Spratling, 2008, 2014).

In this work, we investigate learning and adaptation mechanisms in hierarchical cortical systems to develop a functional account for the role of feedback mechanisms. More specifically, we address the role hierarchical feedback may play in the online learning of visual representations. The study builds upon our previous modeling of a generic cortical architecture at the level of cortical columns. Model areas are defined by regular grids of interconnected columns, which are combined to define cortical subsystems, each composed of distributed networks of interconnected areas. Each model column is described at a mesoscopic level considering a compartmental structure that subdivides a cortical site into an input stage of specific signal filters, as well as superficial and deep layers as columnar compartments. Within this framework, feeding input signals drive the activity of columns and their lateral interactions. Feedback signals are thought to act in a modulating fashion so that responses at higher level cortical stages alone cannot generate activations in earlier representations (thus implementing a no-strong-loops principle; Crick and Koch, 1998). However, we demonstrate that interaction between different groups of cells allows to segregate the feedback signal strength that modulates the feedforward input activation such that the strength of feedback could be traced to serve as a signature how the expectations or predictions converge to the activation distribution of the driving input. The feature specificity of neurons in a cortical column is established through a learning mechanism that evaluates correlative activation in a scheme of modified Hebbian weight adaptation (Grossberg, 1988). During learning the connection strengths of bottom-up weights (to propagate converging driving input signals) are adapted. The applied learning scheme imposes a constraint such that the weights conserve their total energies so that variable input that is distributed over a population of neurons in columns does not lead to any bias in the incremental input segmentation. Thus, segmentations are allowed to build different and partly overlapping categorical patterns in which the total energy of the bottom-up input weights is normalized. The recurrent feedback from higher level representations generates a prediction, which consists of a pattern of the expected input activation, that drives the receiving representation of a column best. For that reason, the modulatory top-down feedback connections are here learned by using a slightly different weight adaptation mechanism. The feedback weights define a top-down projective field, which represents the expected average input activity distribution of the cell. Taken together, feedforward learning enables the generation of prototypical form pattern representations, whereas feedback weights encode the characteristics of the category a stimulus is currently assigned to by the visual system. Thus, feedback and feedforward learning in combination realize an online associative memory mechanism, allowing the separation of an input stimulus and an according prototypical representation (see Carpenter, 1989). Using a modulation mechanism, the differences between an input pattern and an internal category representation are amplified in the input signal, yielding category building, consolidation, or refinement. The framework thus defines an important building block for the automatic incremental learning of visual categories (at different stages in the visual hierarchy). The compartmental structure and the neuronal interactions allow to stabilize the learning to prevent oscillatory learning as well as effects of overshadowing existing representations, connoted as the plasticity-stability dilemma (Grossberg, 1988). Using simple form patterns as input stimuli, we demonstrate that the model allows to automatically distinguish and refine the encoding of overlapping patterns and to trigger the learning of new categories when the input patterns differ significantly.

## 2. Generic model architecture

### 2.1. Overview of the model components and functional architecture

**Figure 1:**
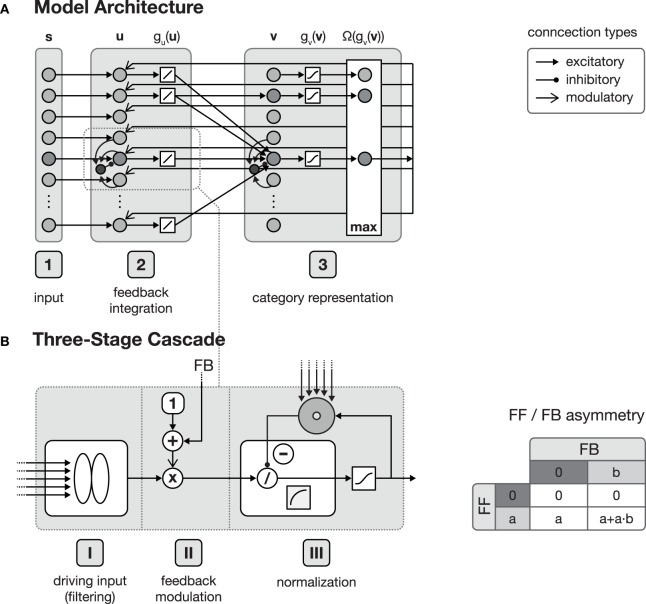
**Model architecture**. **(A)** shows the overall structure of the proposed model, which is composed of interconnected cortical columns, subdivided into a compartmental structure of three processing layers. The first layer propagates input activities **s** to the second layer, where they are combined with a residual signal derived from feedback activities emitted from layer 3 and the current activity *g*_*u*_(**u**). After normalization, layer 3 category cells perform a correlation of the current layer 2 activities and their respective synaptic input weights. The cell with the strongest activation *g*_*v*_(**v**) is then selected by a winner-take-all (WTA) mechanism for weight adaptation and activity propagation. In principle all of the model layers consist of a three-stage processing cascade as illustrated in **(B)**. The cascade comprises an initial input filtering (stage **I**), the modulation of the activity (stage **II**) and a final pool normalization (stage **III**). Re-entrant feedback from higher level areas is incorporated in stage **II** where the current activity is modulated by (1 + *net*_*FB*_). This kind of feedback integration is essential, since it results in an asymmetry of the roles the feedforward and the feedback signal play in the signal processing. As illustrated in the table on the right-hand side of **(B)**, without the presence of a feedforward signal, a feedback signal cannot evoke any activity.

The function of the proposed network architecture has been discussed in the previous section in order to motivate key aspects of automatic acquisition of shape and object representations and how underlying cortical structural principles and mechanisms might contribute to its realization. In this section we present formal model mechanisms as as a sketch of how the processing might be implemented dynamically. The basic structure of the generic model architecture is defined by three layers, each of which consisting of sheets of mutually interconnected computational elements (see Figure 1). These layers in the model roughly correspond to areas in cortex. Henceforth, we will address these stages by calling them layers or areas, given the particular context in the text. In the three layer architecture, the input layer is sketched like a simple replica of the input field fed by the current stimulus. The inclusion of such an explicit layer implicitly states that it may represent the result of some complex preprocessing that transforms the raw input into activity distributions referring to certain feature dimensions represented in a distributed fashion in (visual) cortex. As the same structure and composition of abstract columns can be replicated and more fine-tuned at different levels of cortex-like processing, we suggest that the outlined model architecture is generic in its structure and function. The computational elements in layers two and three both consist of an abstract model representation of cortical columns. Each of such columnar units itself is organized in a cascade of three processing stages: (I) input filtering, (II) activity modulation, and (III) pool normalization (details of the functional properties are discussed in, e.g., Neumann and Sepp, 1999; Bouecke et al., 2011; Brosch and Neumann, 2014a,b). These cascade stages roughly correspond to the division of cortical areas, with their six layers (Lui et al., 2011), considering the layer of terminating bottom-up input, as well as the superficial and the deep layers of cortex (Self et al., 2013). Each of these stages is represented by a model neuron that itself is a single-compartment dynamic element with gradual activation dynamics representing the average potential of a group of mutually coupled neurons. A firing-rate function *g*( · ) converts the potentials into an output activation. Feedforward and feedback signal streams are combined at the level of individual columns (Larkum, 2013; see Brosch and Neumann, 2014a for a model implementation). In the proposed architecture, the second layer combines the input multiplicatively with a residual signal that is derived from the current input pattern and a feedback signal emitted from the successive layer 3 which is biased by a tonic activity level (Eckhorn, 1999; Neumann and Sepp, 1999). Thus, the feedforward signal gates the re-entrant top-down signal so that the gain of existing activity can be increased by matching feedback signals. Feedback signals alone, however, cannot generate any activation for void bottom-up signal input. The feedback signal is generated here by a residual template, which contains the difference between the expected input (of the winning category node) and the current bottom-up input signal. As long as the difference does not vanish, the feedback mechanism leads to an increase in the activity gain of the current input. This mechanism deviates from the scheme described in e.g., (Bouecke et al., 2011), where the top-down signal is used instead of the residual signal. However, the dynamic properties of the non-linear circuit are retained.

Apart from the rather detailed network structure for generating an activation dynamic, the bidirectionally coupled network architecture is capable to adapt its connection weights, and is thus able to learn new category and subcategory representations as well as the expected average input distributions that have been established to drive a specific target category representation. In layer 3 of the generic architecture, category and subcategory representations are established using Hebbian learning mechanisms. Here, two complementary synaptic weight distributions are learned, each serving a different purpose within the proposed network. The feedforward synaptic weights are intended to build the category and subcategory representations during training, whereas the feedback weights are used to propagate an internal representation of the currently best matching category back to layer 2. This allows the estimation of the difference between the current input and the category assigned to the input after the feedforward sweep. Thus, layer 2 cells are able to combine the input with the derived difference signal and potentially evoke the activation of a different category/subcategory cell at the level of layer 3.

We split our presentation of the detailed model components into two major parts. First, we describe the activation dynamics, i.e., the formal definition of the generation of activities in each model computational element along the structure outlined in the previous paragraph. The activations are dependent on the input, the weightings of the spatial couplings for the input, and the current state, or activation of a model neuron. We emphasize how the incorporation of top-down feedback signal pathways can achieve rich and stable computations in such a network architecture. Second, in order to automatically acquire behaviorally relevant feature and category representations, the system can learn by adapting the weightings of the connection patterns between the model areas. We describe the weight, or learning, dynamics separately by focusing on the formal description of the weight adaptation and their key functionality. We finally link activation and learning dynamics to emphasize the capability of such building blocks for autonomous learning in cortical architectures.

**Figure 2:**
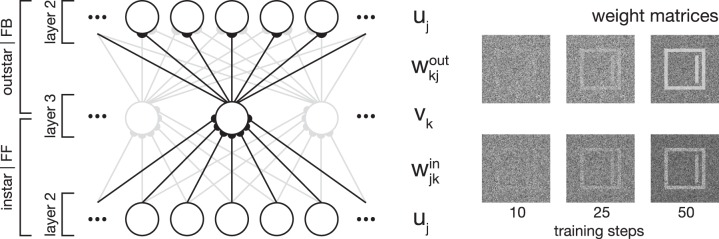
**Network plasticity**. Model layer 3 cells establish categorial and subcategorial representations using Hebbian learning in combination with a modulatory feedback mechanism. As shown on the left-hand side, they realize an associative memory by combining instar and outstar learning schemes. The afferent connections weights *w*^*in*^ are used to select the best matching representation to a given input. The weights projecting away from the cell *w*^*out*^ are incorporated in the top-down feedback to layer 2 cells. For a given stimulus, only the cell with the highest activation is selected for weight adaptation. On the right-hand side, exemplary weight matrices are shown after several training steps. The matrices were obtained during the simulation of Experiment 1 (see Section 3.1).

In essence, category and subcategory learning is enabled using two complementary core mechanisms. First, an associative memory is realized through the combination of an instar with an outstar learning scheme (compare Carpenter, 1989; see Figure 2). This allows the assignment of a given input to the currently best matching internal representation, as well as the propagation of the corresponding feedback pattern to re-enter at an earlier processing layer. Second, the differences between an input signal and the pattern associated with the best matching internal representation of the input define the modulatory signal to enhance the gain of the bottom-up feedforward signal.

In the following, we first describe the overall properties of the three-stage processing cascade, which forms the generic building block for all of the model layers.

### 2.2. Activation dynamics

#### 2.2.1. Three-stage processing cascade

The first stage of the model cascade performs a linear filtering of the input. To model the response *r* of a cell, we calculate the weighted sum on the input to a cell, as defined by

(1) $$r=\sum_{j\;=\;1}^{N}K_{j}\;\cdot \;s_{j},$$

with *N* the number of input cells with activities **s**, which are modulated by the weight distribution **K**. Within the proposed model, the filtering step either results in the propagation of the impulse response to a given input (for layer 2 cells) or **K** corresponds to a weight distribution derived from the input statistics (for layer 3 cells, see Section 2.3.1).

At the second stage of the cascade, responses from the previous filtering are modulated by re-entrant input from higher-level model areas. Modulation is thereby performed in a way, such that only existing activities in an input signal can be amplified (and thus activities cannot emerge solely provoked by a feedback signal). With *r* being the unmodulated driving signal and *net*_*FB*_ being the strength of the feedback signal, the modulated response of a cell is given by

(2) $$r_{FB}\propto r\;\cdot \;(1+net_{FB}).$$

This kind of feedback incorporation assures that if *r* = 0 no signal is generated as output, independent of the strength of the feedback *net*_*FB*_. On the other hand, the input signal *r* is left unchanged in the absence of any feedback signal (i.e., *net*_*FB*_ = 0, see Figure 1B).

Prior to the final stage of the processing cascade, we apply a transfer function to convert the responses into a cell activation level. For simplicity we employ a linear transfer function at layer 2 of the proposed model, whereas at layer 3, a non-linear sigmoidal transfer function is used.

At the final stage of the processing cascade, activity normalization through divisive mutual inhibition within a pool of neurons (shunting inhibition) is applied. In its dynamic formulation, the rate change of the a signal *r*^*norm*^_*j*_ depends on the current activation level *r*_*j*_ and the amount of inhibitory input activation in the pool *q*_*j*_

(3) $${\dot{r}}_{j}^{norm}=-\alpha_{r}\;\cdot \;r_{j}^{norm}+\beta_{r}\;\cdot \;r_{j}-r_{j}^{norm}\;\cdot \;q_{j}$$

(4) $${\dot{q}}_{j}=-q_{j}+\;\cdot \;\sum_{k\;=\;1}^{M}r_{k}\;\cdot \;\Lambda_{jk}^{pool},$$

with *M* denoting the size of the incorporated population in the neighborhood of location *j* and the weighting function Λ^*pool*^_*jk*_. The constant β_*r*_ controls the scale of the normalized signal, α_*r*_ denotes the passive decay rate.

In the following, we first describe the forward sweep throughout the proposed model layers. After the functional differences between the different model layers have been described in detail, we will emphasize the feedback connections and their role for the task of category and in particular subcategory learning.

#### 2.2.2. Model layer 1/2

Layer 1 and layer 2 follow a pairwise connection scheme, such that each input cell in layer 1 is only connected to exactly one cell in layer 2 (see Figure 1). At the level of layer 2, the linear filtering step described in Equation (1) is equal to an identity function. Thus, the response of a layer 2 cell is defined by the following equation:

(5) $${\dot{u}}_{j}=-\alpha_{u}\;\cdot \;u_{j}+\beta_{u}\;\cdot \;s_{j}-u_{j}\;\cdot \;q_{j},$$

where *s*_*j*_ denotes the output of a layer 1 cell, *u*_*j*_ describes the layer 2 cell response which relates to the membrane potential of real cells (*j* denoting the cell position). The constant α_*u*_ denotes the passive decay rate, whereas β_*u*_ describes the input scaling factor. The potentials are converted into an activation level, or firing rate, by the transfer function *g*_*u*_(*u*_*j*_) (see Brosch and Neumann, 2014a for a formal specification and analysis). Here, we employ a linear transfer function with rectification such that no negative responses occur,

(6) $$g_{u}(u_{j})={\left[u_{j}\right]}^{+},$$

with [*u*]^+^ = *max*(*u*, 0). The competitive interaction against a pool of cells to accomplish activity normalization is defined as

(7) $${\dot{q}}_{j}=-q_{j}+\sum_{k\;=\;1}^{N}g_{u}(u_{k})\;\cdot \;\Lambda_{jk}^{pool},$$

with *N* denoting the size of the incorporated population in the neighborhood of location *j*, weighted by Λ^*pool*^_*jk*_. Without the incorporation of any feedback signals, layer 2 cells solely perform an activity normalization on the output activities **s** of layer 1 and propagate the result to layer 3.

#### 2.2.3. Model layer 3

Layer 2 and layer 3 cells form a complete bipartite connection graph with connections in both directions (see Figure 1), with corresponding synaptic coupling strengths *w*^*in*^ for feedforward and *w*^*out*^ for feedback connections. The output of layer 2 *g*_*u*_(**u**) is filtered by the feedforward weights *w*^*in*^_*ji*_ to generate the strength of the response *v*_*i*_ of a layer 3 cell, which finally enters a competition with the surrounding pool activation (**u** denoting the field of input activities represented as a vector), as defined by:

(8) $${\dot{v}}_{i}=-\alpha_{v}\;\cdot \;v_{i}+\beta_{v}\;\cdot \;\sum_{j\;=\;1}^{N}g_{u}(u_{j})\;\cdot \;w_{ji}^{in}-v_{i}\;\cdot \;q_{i},$$

with the passive decay rate α_*v*_ and the input scaling factor β_*v*_. The response is then converted into an activity level using the non-linear sigmoidal transfer function *g*_*v*_ with the parameters κ_*log*_ (steepness) and μ_*log*_ (mean response level),

(9) $$g_{v}(v_{i})=\frac{1}{1+e^{\kappa_{\log}\cdot (\mu_{\log}-v_{i})}}.$$

As in layer 2, the final competition for activity normalization is defined by a non-linear competition of target activity and the integrated activation over a pool of neurons, which is determined by

(10) $${\dot{q}}_{i}=-q_{i}+\sum_{k\;=\;1}^{M}g_{v}(v_{k})\;\cdot \;\Lambda_{ik}^{pool},$$

with *M* denoting the number of cells in layer 3 and the weighting function Λ^*pool*^_*ik*_.

### 2.3. Network plasticity

In the previous part we have briefly introduced the formal description that covers the activation dynamics of the model mechanisms in the suggested generic architecture. As already mentioned, the architecture consists of three layers that roughly correspond to model areas of visual cortex. As outlined in Figure 1, the first area represents the input, that can be the raw responses of preprocessing the input directly (like in the early stages of the visual hierarchy, e.g., V1 and V2) or the output responses from a cascade of already more sophisticated processing to build intermediate level representations (like in the higher stages of the visual hierarchy, e.g., V3 and V4). The second and third model areas in the model layout are connected bidirectionally representing feedforward and feedback sweeps of signal propagation in cortex (Lamme and Roelfsema, 2000). We have already explained how the two counterstream signal flows converge to build representations of integrated bottom-up evidences (from signal processing) and top-down predictions or expectations (generated by higher level stages of category representations). In this part we equip the network architecture with mechanisms of adapting the connections to learn representations in specific input weights. We suggest here that learning occurs along the feedforward as well as the feedback pathways (an outline of the learning architecture is shown in Figure 2). The functionality behind such a, again generic, principle is that feedforward connections learn weighting profiles that increase the probability for an input activation pattern to generate amplified responses in the recipient unit. Likewise, learning of feedback connections is intended to build up a representation in which source node activations (at the higher-level stage of the architecture) will generate a distribution of (pre-) activations as the expected average activity at the input stage that drives the node. The expectation is thus represented in the top-down connection weights (see Layher et al., 2014 for a model learning architecture that follows the same generic principles). Here, we develop a mechanism with a slightly different emphasis. The network aims to develop categories and also (later) to advance the automatic establishment of subcategories driven by significant local deviations of the already existing category representation. Therefore, the signal that is carried by the top-down feedback connections needs to be transformed into a residual signal such that the difference from the expected activation pattern is registered. We suggest that such residual patterns are generated at the neuronal *activation* pattern, instead of the weighting pattern.

In the following, we present the formal descriptions of the mechanisms used for the weight adaptation. We also briefly sketch how these relate to achieve the target representations for the desired bottom-up and top-down processing. The adaptation of the connection weights, for both feedforward and feedback, can be considered for individual neuronal sites in layer 3: The *receptive* field, or fan-in structure, is defined for connections along the bottom-up signal transmission that converge on a target neuron, *u* → *v*. The *projective* field, or fan-out structure, on the other hand, is defined for connections along the reverse direction that spread out from the target neuron back to the previous stage, *v* → *u* (compare Carpenter and Grossberg, 1987b; Lehky and Sejnowski, 1988 for discussions of the underlying function of such connection principles). The activity dependent adaptation rules of such connection weights, namely feedforward, *w*^*in*^ and feedback *w*^*out*^ weights, are governed by modified versions of Hebbian correlation learning principles (Hebb, 1949). These modifications lead to stability and proven convergence properties and it can be shown that the learning rules optimize some target functionals.

The target neurons at layer 3 (with the adaptable fan-in and fan-out connections) are considered here to represent categories in a classification or recognition mechanism. For simplicity, we consider learning by weight adaptation that is allowed only for the category node that is maximally activated, as in many other related learning paradigms (e.g., Kohonen, 1982; Carpenter and Grossberg, 2003). Such a model neuron is selected by a simple maximum selection operation, or winner-take-all (WTA) mechanism (Grossberg, 1973) and the weight adaptation is triggered subsequently,

(11) $$\Omega (g_{v}(v_{k}))=\{\begin{array}{ll} 1 & \text{if }k=\underset{i\;=\;1\ldots M}{arg\;max}g_{v}(v_{i})\\ 0 & \text{otherwise}. \end{array}$$

It should be noted that the WTA selection is chosen here for simplicity. As an alternative, one could use a softmax mechanism as well (e.g., Roelfsema and van Ooyen, 2005), without changing the overall functionality of the approach. The specific learning rules for feedforward and feedback connections are presented below.

The learning of the feedforward weights *w*^*in*^, as well as the feedback weights *w*^*out*^ is realized using Hebbian learning principles, which are described in the following.

#### 2.3.1. Learning of feedforward connections

We utilize a variant of Hebbian correlation learning which prevents the changes of connection weights to grow without bounds. The stabilization is here achieved by a forgetting term that reduces the weight proportionally to the postsynaptic cell activation. The weight change for the receptive fields is formally defined by

(12) $${\dot{w}}_{jk}^{in}=\Omega (g_{v}(v_{k}))\;\cdot \;\eta_{in}\;\cdot \;g_{v}(v_{k})\;\cdot \;(g_{u}(u_{j})-g_{v}(v_{k})\;\cdot \;w_{jk}^{in}).$$

The r.h.s. of the equation is defined by the switch Ω( · ) to enable/disable neurons for adaptation of their weights and a learning rate η_*in*_. The extended Hebbian correlation term is defined by *g*_*v*_(*v*_*k*_) · (*g*_*u*_(*u*_*j*_) − *g*_*v*_(*v*_*k*_) · *w*^*in*^_*jk*_). In other words, the learning is gated by the activation of the postsynaptic neuron. Here, the Hebbian term *g*_*u*_(*u*_*j*_) · *g*_*v*_(*v*_*k*_) is combined with the forgetting term *g*_*v*_(*v*_*k*_)^2^ · *w*^*in*^_*jk*_ to balance the temporal change and bound the growth of the cell's synaptic input weights. It has been demonstrated that such a learning mechanism extracts the first Eigenvector of the input distribution (Oja, 1982, 1992). Another property of the Oja learning rule is of even more interest here: The learning of the bottom-up feedforward weights approaches a fan-in connection pattern in which the weight energy is conserved (Dayan and Abbott, 2005). The fan-in weight vector **w**^*in*^_*k*_ is adapted over time to reach equilibrium, such that lim_*t*→∞_ **ẇ**^*in*^_*k*_ = *v*_*k*_ · **u** − γ *v*^2^_*k*_ · **w**^*in*^_*k*_ = 0 (with γ as a positive constant value that scales the balancing component). The equilibrium weight energy is then

(13) $$\|w_{k}^{in}{\|}^{2}=\frac{1}{\gamma }.$$

Assuming γ = 1 we get a unit length for the input weights to single category nodes. This, in turn, prevents input activation distributions to bias the output activity at the category representation, given that the input activity distribution is normalized as well. The latter property is achieved by the normalization stage of the pool interaction defined in the activations dynamics of the network stages above.

#### 2.3.2. Learning feedback connections

Again, we utilize a stabilized Hebbian weight adaptation formalism. In its dynamic formulation, the weight changes for projective fields is formally defined by

(14) $${\dot{w}}_{kj}^{out}=\Omega (g_{v}(v_{k}))\;\cdot \;\eta_{out}\;\cdot \;g_{v}(v_{k})\;\cdot \;(g_{u}(u_{j})-w_{jk}^{out}).$$

As for the adaptation of the receptive field, or fan-in, weights (Equation 12) we utilize the switch Ω( · ) to enable/disable weight adaptation and a learning rate η_*out*_ for the projective, or fan-out, weights. The extended Hebbian term is here defined by *g*_*v*_(*v*_*k*_) · (*g*_*u*_(*u*_*j*_) − *w*^*out*^_*jk*_). The learning is gated by the activation of the neuron that represents the category, which is presynaptic to the projective field considering the representation generated for the top-down feedback connections. Unlike the learning rule discussed in Equation (12), the forgetting term to balance the temporal change is controlled by the weight only. Such a weight adaptation mechanism defined in Equation (14) has been suggested for gated steepest descent learning in long-term memory formation, e.g., in Adaptive Resonance, or ART networks (Grossberg, 2013b). The adaptation of the fan-out weight vector **w**^*out*^_*k*_ over time reaches equilibrium, such that lim_*t*→∞_ **ẇ**^*out*^_*k*_ = *v*_*k*_ · **u** − γ *v*_*k*_ · **w**^*out*^_*k*_ = 0 (with γ as a positive constant value that scales the balancing component). The equilibrium weight energy is then

(15) $$w_{k}^{out}=\frac{1}{\gamma }u.$$

Assuming γ = 1 we achieve a projective field, or fan-out, pattern for the connection weights corresponding to the (average) expected input activation represented in **u**. Activation of a category node, thus, biases the receiving postsynaptic model neurons according to the predicted pattern the category expects to receive for its best tuning input. Feedback learning may also utilize the learning rule of Oja as for learning the feedforward connections described above. In this case the weight distribution of the projective field would converge to the first Eigenvalue of the expected input, instead of its mean. We have tested this and observed similar network performance. The latter implementation argues in favor of symmetric learning mechanisms for bottom-up and top-down connection weights. We decided to use a version in which the feedback projections approach the expected average input activation that represents the tuning of the individual categories, as in Equation (15).

### 2.4. Feedback for subcategory learning

The mechanisms presented so far contributed to the feedforward as well as a generic feedback sweep of the model. The feedback sketched so far generically considered the modulatory influence a feedback signal has on any feedforward input representation. The mechanism emphasized the symmetry breaking property in which bottom-up signals gate the activity generation (at stage 2 of the processing cascade described in Section 2.2.1) which can be selectively amplified by the presence of matching feedback signals. Here, without incorporating the feedback from layer 3, the learning rules defined in Section 2.3 would successfully learn representations of input categories, but without the potential of further refining them on a subcategorial level. As stated earlier, the feedback allows the estimation of the difference between the current input and the category assigned to the input after the feedforward sweep. Thus, layer 2 cells are able to combine the input with the derived difference signal. If the difference and the modulation strength after the feedback sweep is large enough, learning is potentially triggered such that an associated new subcategory is built using a so far unused layer 3 cell. The enhancement of the layer 2 responses by modulating feedback changes (Equation 5) to

(16) $${\dot{u}}_{j}=-\alpha_{u}\;\cdot \;u_{j}+\beta_{u}\;\cdot \;s_{j}\;\cdot \;(1+\lambda \;\cdot \;res_{j}^{templ})-u_{j}\;\cdot \;q_{j},$$

where *res*^*templ*^_*j*_ denotes the residual signal derived from the feedback *net*^*FB*^_*j*_ of the best matching category cell [selected by Ω(*g*_*v*_(*v*_*k*_))] and the current activity *g*_*u*_(*u*_*j*_). λ is controlling the influence of *res*^*templ*^_*j*_ on *u*_*j*_ and thus is crucial for the extent of the difference between a modulated input and a category assigned in the feedforward sweep. The residual signal *res*^*templ*^_*j*_ is defined by

(17) $$\begin{array}{l} res_{j}^{templ}={\left[g_{u}(u_{j})-net_{j}^{FB}\right]}^{+}\\ \;={\left[g_{u}(u_{j})-\Omega (g_{v}(v_{k}))\cdot w_{kj}^{out}\right]}^{+}, \end{array}$$

with [*x*]^+^ = max(0, *x*) denoting a rectification operation limiting *res*^*templ*^_*j*_ to positive values. A closer look at the presented model dynamics may help us to reveal the potential roles that feedback plays in the context of category learning. According to Equation (17), the feedback signal acts as a predictive coding scheme, since *net*^*FB*^_*j*_ expresses what the model expects how an input of a given category looks like on average. On the other hand, the expression *s*_*j*_ · (1 + λ · *res*^*templ*^_*j*_) in Equation (16) realizes a biased competition mechanism, favoring input components, which are in accordance with the residual signal *res*^*templ*^_*j*_. In essence, this kind of feedback incorporation results in an amplification of the differences between the currently best matching internal representation and the input. During learning, the difference between a category representation and individual instances of the category increases with the number of stimuli of the same category. If the effect of this difference on the input is large enough, a new subcategory representation is established.

## 3. Results

**Figure 3:**
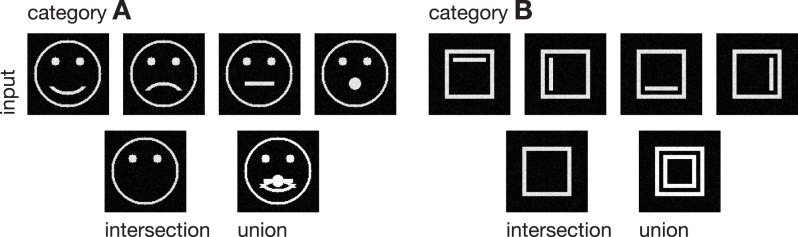
**Input stimuli**. Two different categories of stimuli were used as input to the model, each with a set of subcategories. Category **A** is composed of four pictographical images of a face, only differing in the shape of their mouth. Category **B** consists of four variations of a square inclosing an either vertically or horizontally oriented bar at different positions. The bottom row shows the intra-category union and intersection for both categories, pointing out the differences and similarities in each category.

In the following, we demonstrate the capabilities of the proposed model in learning category and subcategory representations using two categories of artificial input stimuli. As shown in Figure 3, category **A** contains four variations of a pictographical face. Category **B** is composed of four squares inclosing an either vertically or horizontally oriented bar at different positions. Without the loss of generality, we used very simplified stimuli to keep the computational complexity and in particular the necessary preprocessing steps as simple as possible. This allows us to keep the focus strictly on the role which feedback might play in the task of category and subcategory learning. The stimuli were generated with the dimensions of 100 × 100 *px* with intensity values ranging from 0 to 1. The number of input units in layer 1 thus is always 100 · 100 = 10000 units. As mentioned in Section 2, the cells in layer 1 and those in layer 2 follow a pairwise connection scheme, so that layer 2 consists of the equivalent number of 10000 units. The number of layer 3 cells differs from experiment to experiment. Note that in all experiments, there remained at least one unused layer 3 cell after training, which was never selected for weight adaptation. Thus, the number of units in layer 3 never was a limitation to the establishment of a new category or subcategory representation. During training, Gaussian noise with mean μ = 0 and a standard deviation of σ = 0.05 was added on each of the input stimuli, with values clipped to the range of [0, 1]. If not stated otherwise, we used learning rates of μ_*out*_ = μ_*in*_ = 2^−4^ and a feedback gain factor of λ = 2^5^. These values were found to be a suitable balance between the learning speed and the influence of the feedback. The parameters of the logistic function as defined in Equation (9) were set to μ_*log*_ = 700 and κ_*log*_ = 0.0075, such that the transfer function results in a mean activation level of *g*_*v*_(700) = 0.5 when roughly half of the input energy of one of the used stimuli is present in the input signal. The weights *w*^*in*^ and *w*^*out*^ of the category cells at model layer 3 were initialized with random values drawn from a normal distribution with mean μ = 0.75 and a standard deviation of σ = 0.1, allowing empty category cells at layer 3 to be activated by just a small number of active input cells.

For the ease of computational complexity, we simulate the dynamics described in Section 2 using the corresponding steady-state equations. An in depth analysis of the activation dynamics can be found in Brosch and Neumann (2014a). Within the simulations, one (training) step—or iteration—corresponds to the presentation of one input stimulus, consisting of one feedforward and one feedback sweep through the model. Activities of the layer 3 cells are evaluated after the feedforward and after the feedback sweep and both trigger the adaptation of a categorial and/or subcategorial representation.

In total, we performed four experiments, each highlighting on a different aspect of the proposed model and learning mechanisms. In the first experiment, we show in principle how the model successfully learns a representation of a category of visual input stimuli and decomposes the category into subcategories. The second experiment is intended to demonstrate the invariance of the proposed learning mechanism to the order in which the stimuli are presented. Experiment 3 focuses on the importance of the feedback signal for the task of subcategory learning by contrasting Experiment 2 with a nearly identical experimental setup. The sole difference to Experiment 2 is that the incorporation of feedback is suppressed by setting the feedback gain parameter λ to λ = 0. In the last experiment we demonstrate how the model generalizes across the number of categories present in the input data and show how it successfully establishes representations for two categories of visual input and their subcategories.

All simulations were carried out using Mathworks Matlab R2014a.

### 3.1. Experiment 1

**Figure 4:**
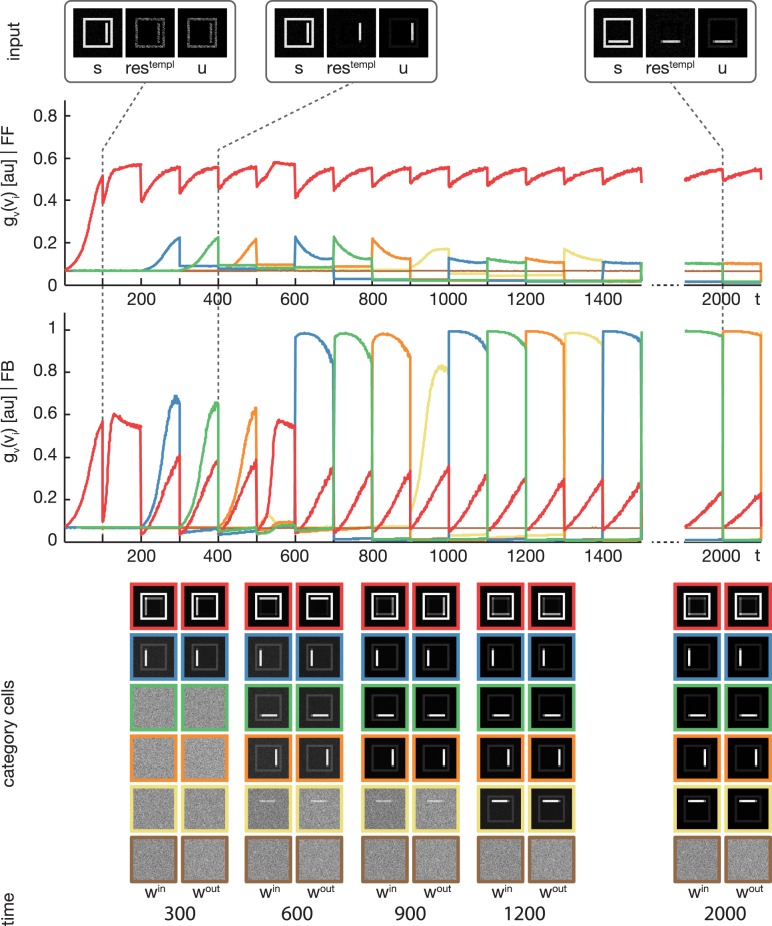
**Experiment 1**. The model was trained using four rectangular shapes (see Figure 3, category **B**) as input stimuli. Stimuli were presented in sorted blocks. Six category cells (color-coded) were initialized with random weights. The first row shows exemplary input configurations **s**, along with the corresponding residual signal **res**^**templ**^ and the input signal **u** after feedback modulation. In the second row, the activities of the six category cells before the feedback sweep are shown. As can be seen, before the feedback is effective on the input, only one cell (encoded in red) responds to all input configurations. This cell represents the overall category cell. The second row shows the activities after the feedback sweep. In the last row the corresponding category cell weights are displayed framed by colors according to the activity plots. It can be seen, that in the beginning all inputs are learned into one category cell. After about 200 training steps, the effect of the feedback is high enough to trigger the learning of a new subcategory representation. This process repeats several times, until each subcategory is represented by an own category cell.

We trained the proposed model using the rectangular stimuli of category **B** as shown in Figure 3. The stimuli were presented in epochs of four blocks of sorted stimuli, each block containing 100 instances of one of the four rectangle variations. At model layer 3, six cells were used during the training. To slow down the weight adaptation process and highlight on the establishment of new subcategory representations, we used a learning rate of μ_*out*_ = μ_*in*_ = 2^−5^, set μ_*log*_ to 800 and initialized *w*^*in*^ and *w*^*out*^ with random values drawn from a normal distribution with μ = 0.5 and σ = 0.1. The activities of the layer 3 cells after the feedforward and the feedback sweep are shown in Figure 4 along with the corresponding weights *w*^*in*^ and *w*^*out*^ after several training steps. Over the first training steps, the model develops a combined representation of the first and the second rectangular shape containing information about the surrounding rectangle, as well as portions of information about the interior of the two shapes. After 200 training steps, the effect of the learning mechanism starts to be twofold. After the feedforward sweep, the overall category representation is adapted to the current input stimulus. On the contrary, after the feedback sweep a subcategorial representation is learned by recruiting an additional layer 3 cell. The effect of the feedback signal now is large enough to suppress the outer rectangular shape and highlight on the differences between the overall category representation and the current input stimulus. This process continues until all of the four input variations are represented in an own subcategory cell. After learning, the feedforward sweep always results in a high activation level *g*_*v*_(*v*_*i*_) of the overall category cell that represents the generic shape (refer to the second row of Figure 4). After the feedback sweep, however, the subcategory cell representing the specifics of the particular input stimulus is the one with the highest activation level.

### 3.2. Experiment 2

**Figure 5:**
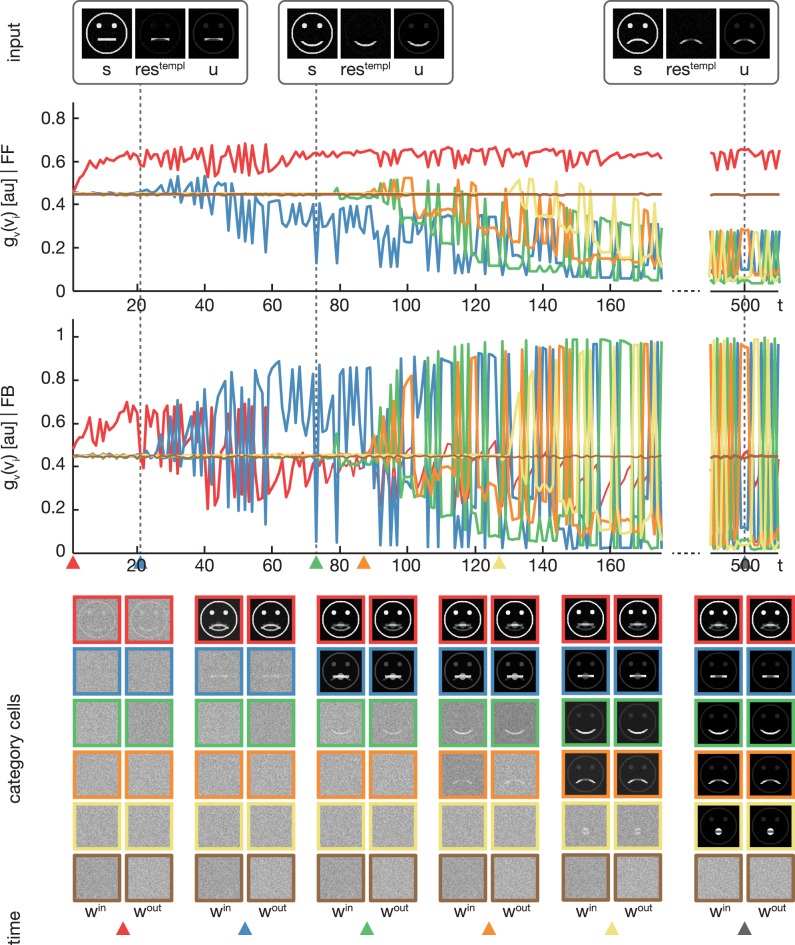
**Experiment 2**. In the second experiment, we trained the model using four variations of pictographical faces (see Figure 3, category **A**). In contrast to Experiment 1, stimuli were presented in random order. The display of the results is organized as in Figure 4. In addition, colored triangles indicate the points in time, when a category or subcategory cell was selected for weight adaptation the first time. Although the stimuli were presented in random order, the model successfully separates the input stimuli into subcategories.

In the second experiment, the proposed model was trained using the pictographical faces of category **A** (see Figure 3) as input. Stimuli now were presented in random order. As in Experiment 1, six category cells at model layer 3 were used. All training parameters were set to their default values (see Section 3). Figure 5 shows how category and subcategory cell representations are learned during the simulation. Again, the residual signal *res*^*templ*^ increases with the distinctiveness of the already established category representation and thus the effect of the feedback signal increases. Already after 21 training examples, the difference between the current input and the existing category cell is high enough to yield a modulation of the input effective-enough to evoke the establishment of a new subcategory. This process repeats several times, since after 127 learning iterations all of the variations of category **A** are represented in an own subcategory cell. Altogether, the model successfully learns category and subcategory representations, even though the stimuli are presented in random order.

### 3.3. Experiment 3

**Figure 6:**
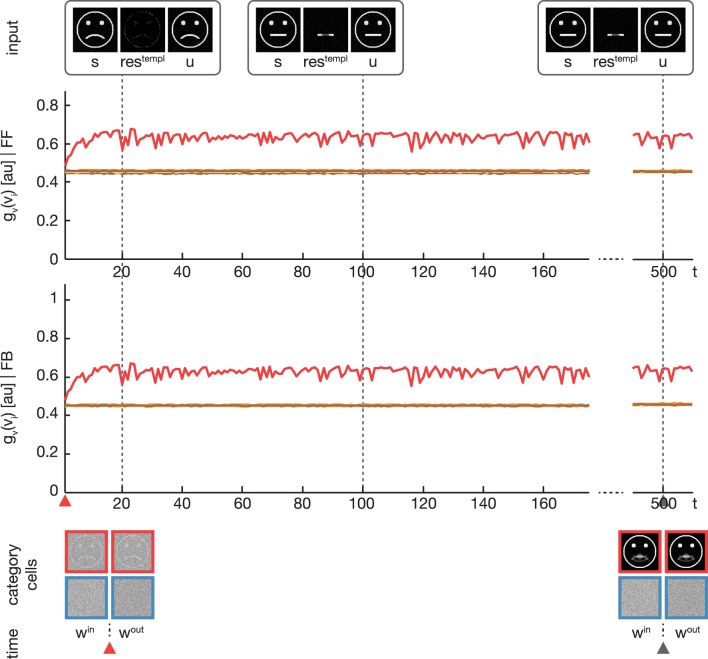
**Experiment 3**. The simulation was performed with the same setup as in Experiment 2. Only the feedback gain parameter λ was adjusted to λ = 0, disabling any influence of the feedback on the learning. As expected, the activation levels *g*_*v*_(*v*_*i*_) before (second row) and after (third row) the feedback sweep are identical. Without the feedback modulation, no subcategory representations are learned and only one overall category representation is established.

In a third experiment we conducted a simulation equivalent to the one in the second experiment, but now with disabling the feedback signal by setting λ = 0 (see Figure 6). As expected, without the feedback signal no subcategory representations are established and just one overall category representation is learned.

### 3.4. Experiment 4

**Figure 7:**
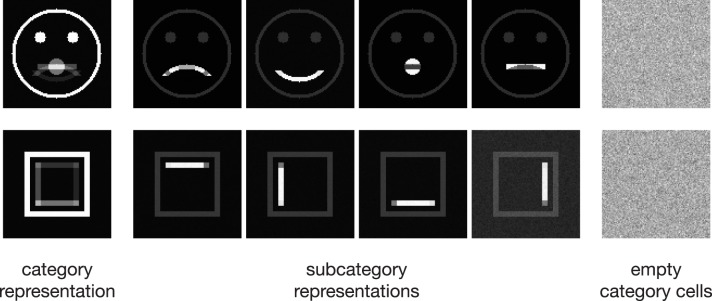
**Experiment 4**. Both stimulus categories shown in Figure 3 were used as input to the model. The distributions of the input weights *w*^*in*^ are shown after 1000 training steps for twelve category cells at model layer 3. The model successfully established representations for all input variations of both classes and two overall category representations, one for each input category.

For the last experiment we used both categories **A** and **B** shown in Figure 3 as input stimuli. The parameters were equivalent to those described in Experiment 1 but now twelve category cells at layer 3 were initialized. Since the differences between the two types of stimuli (circular and rectangular) are already large enough before the feedback takes place, the model establishes two overall category representations and successively builds subcategories to these two categories. Figure 7 shows the weights of the established two category, as well as the respective four subcategory cells after 1000 learning steps.

## 4. Discussion

In this work we proposed a hierarchical architecture of cortical feedforward and feedback processing that builds upon previous work on the modeling of recurrent cortical dynamics (Neumann et al., 2007; Brosch and Neumann, 2014a). Here, we particularly focused on the issue how in such networks feature or category representations could be automatically acquired by unsupervised learning mechanisms, which are seamlessly integrated in the recurrent architecture. The core computational elements assumed are cortical model columns that are abstractly described by a three-stage cascade of processing steps. The same elements have been utilized as generic mechanisms in models of form and motion processing, figure-ground segregation, as well as modeling biological motion perception that fuses segregated form and motion pathways (Neumann and Sepp, 1999; Bayerl and Neumann, 2004; Raudies et al., 2011; Layher et al., 2014). As a specific model feature, we have emphasized the role of feedback that modulates feedforward driving inputs such that their gain is increased dependent on the degree of correlation between feedforward and feedback signal activation. In conjunction with subsequent pool normalization the modulatory feedback sweeps realize a way of biased competition (Girard and Bullier, 1989; Desimone, 1998; Roelfsema et al., 2002; Reynolds and Heeger, 2009). The model now incorporates learning mechanisms to automatically build feature/category representations that are generated by the connection weights through adaptation.^1^ Such learning allows to build representations that adapt their specificity to the statistics of the sensory input patterns.

### 4.1. Summary of contributions

The main contributions of the work presented in the paper are twofold. First, the investigated learning mechanisms occur in the feedforward as well as in the feedback connections. These are driven by bottom-up sensory input and top-down feedback signals to re-enter processing at earlier stages. The latter contain context information that allows to embed local sensory input signals into a larger behavioral context and predictions generated thereof. All this is in the spirit of multi-layer learning networks as discussed in Hinton (2007). In that sense feedforward connections will learn the specific configuration of an (average) appearance of an input feature pattern that the learned category is selectively tuned to. Considering static shape and form input the underlying structural principles are based on the cortical architecture of the ventral pathway with mutual interactions between such distributed representations in different cortical areas (Markov et al., 2014). The feedback connections, on the other hand, also learn by adjusting their weights in order to improve the predicted input pattern that maximally excites the feature/category representation. Second, the top-down feedback learning mechanism combines the modulatory feedback (Girard and Bullier, 1989; De Pasquale and Sherman, 2013) with the concept of top-down predictors that tend to minimize the residual error between feedforward sensory signals and the top-down pattern (Rao and Ballard, 1999; Bastos et al., 2012). The idea behind this concept is that weights will be increased when the predicted pattern and the current input differ. The amount of this gain increase depends on the residual difference between these two patterns. The model defines the basis for more principled investigations how cortical sub-networks that are involved in different tasks might be established. In own previous work (Layher et al., 2014), distributed representations of spatio-temporal patterns in the cortical form and motion pathway were learned for articulated or biological motion perception (Johansson, 1973; Giese and Poggio, 2003). Here, sequence-selective representations were established by learning representations of convergent feedforward responses from form and motion representations. Also top-down weights are learned in which the projective field reaches the two separate pathways of form and motion. The principles proposed in this work now allow to further develop the understanding of how such complex distributed representations can be learned and how average categories are learned together with subcategories for components that deviated significantly from the average category representation.

### 4.2. Relation to previous models of cortical learning of representations

Learning of feedforward networks has been investigated intensively before. Most importantly, the connection weights in multi-layer networks have been trained by using backpropagation to minimize the residual error of expected output given a specific input pattern (Lehky and Sejnowski, 1988, 1990; LeCun et al., 1989). Such approaches require a teacher signal that determines the desired target output. The assumption of a supervisor involved in each teaching trial is biologically unrealistic in general. For that reason, a mechanism that is based on reinforcement learning (Sutton and Barto, 1981; Doya, 2007) has been suggested that combines an unspecific global reward-based reinforcement signal with an attentional signal that is backpropagated from the output layer to allow weight adaptation at those units that have been involved in the stimulus-response mapping in the previous processing of the input signals (Roelfsema and van Ooyen, 2005). Also, learning in hierarchical multi-stage architectures for object recognition has been investigated. Approaches range from random sampling of the input pattern space (Riesenhuber and Poggio, 1999; Serre et al., 2007; Mutch and Lowe, 2008; Serre and Poggio, 2010) to clustering techniques to arrive at sparse representations of the input via additional constraints on the connection weight patterns (Aharon et al., 2006) or auto-encoding that minimizes the reconstruction error of the input (LeCun et al., 1998). Recently, learning in multi-layer networks, so-called deep hierarchical networks (Bengio, 2009), has received renewed interest to build networks with high classification rate performance (LeCun et al., 1990; Hinton et al., 2006). Representations in such networks are learned in a sequential manner by learning the connection weight between pairs of layers, starting from the initial sensory-related level. Once learning converges, the next level connection weights are learned. This procedures is recurrently applied until all connections have been determined. The learning mechanisms are based on gradient descent type, for example, realizing stage-by-stage backpropagation learning. Unlike these proposals, the network mechanism here incorporates bidirectional learning of weights along the feedforward as well as the feedback path. The weight adaptation is based on variants of Hebbian correlation learning. These variants stabilize the growth properties of the input and output weight vectors to the computational elements (model columns) in the architecture. As a consequence, the representations built in the connection patterns have specific interpretations: Along the feedforward path we assume an Oja learning scheme (Oja, 1982, 1992). As a result, the fan-in (or receptive field) weight energy of the total input connections from the previous layer neurons tends to be normalized for feedforward signal filtering. This ensures that different input patterns balance their input weights such they enter any subsequent competition or selection step in an unbiased fashion. Concerning feedback learning, connection weight patterns along the recurrent projection (corresponding to the projective field of a feature or category, Lehky and Sejnowski, 1990) approach the average expected input. In other words, the driving category representation generates a prediction pattern that covers the expected input activation that tends to match the tuning of the representation (Grossberg, 1980).

The proposed architecture is influenced by the conception of adaptive resonance theory (or ART; Grossberg, 1980, 1987; Carpenter, 1989). In a nutshell, learning in ART is organized in stages of feedforward and a feedback sweep processing. During feedforward processing the input signal is weighted by the connection pattern, or filter, between nodes in the feature representation and the category layer. These weightings are initialized by some random values. One category will gain a maximal input from the feature representation activated though the input signal, similar to the feedforward sweep in other networks (Rumelhart and Zipser, 1985), and also in the model proposed in this paper. Similarly, the self-organization of feature maps has also been approached by means of connection weight adaptation in hierarchically organized networks, establishing competitive processes for automatic map formation (von der Malsburg, 1973; Kohonen, 1982). The category that is maximally activated will subsequently suppress all other category representations by recurrent lateral center-surround competition. With supra-linear firing-rate functions such a competitive stage leads to a winner-take-all strategy (Grossberg, 1973). The weightings along the feedforward path can be adjusted to approach the (average) signal features. The feedback connections fed by the winning category node (the projective field) are then allowed to adapt their weights as well so that they approach the input activation distribution. In other words, the feedback connections learn the input that maximally drives the currently activated category node to maintain a match between the input and the expectation the category has about its input patterns it is tuned to (resonance condition). If, instead, any momentary input feature pattern maximally drives a category with a top-down expectation pattern that does *not* match the input, then a mismatch occurs and the combined bottom-up and top-down expectation patterns annihilate. In order to now select another existing category or recruit a new category item, a reset wave is triggered that instantaneously shuts off the winning category that was activated maximally but has a mismatching representation in its projection field. This allows the top-down weights of a newly selected category to adjust in order to now better match the input that is coherent with the expected pattern represented by the active category representation (for recent comprehensive summaries and overviews of the ART principle, see Grossberg, 2013a,b). Discrete implementations of ART networks for pattern recognition have been described for binary as well as continuous input pattern representations (Carpenter and Grossberg, 1987a,b). A more specific reference to possible biophysical mechanisms underlying the recurrent interaction and learning has been described in Carpenter and Grossberg (1990), while Molenaar and Raijmakers (1997) presented a continuous time network implementation.

Several other network architectures use feedback connections that can be adapted through a learning process, e.g., Elman (1990; Hinton et al., 2006; Hinton, 2007; Lazar et al., 2009; Rolfe and LeCun, 2013). While (Elman, 1990) maps temporal feature history into an explicit representation through recurrences, a more recent approach by Lazar et al. (2009) utilizes a reservoir of connected neurons in a large pool to learn representations of temporal patterns. A read-out mechanism maps the internal state trajectories onto units through reduction of state-space dimension and clustering of activities. This recurrent network architecture with spiking model neurons emphasizes different mechanisms in the learning of connections weights, namely a simplified version of spike-timing dependent plasticity (STDP; Gerstner et al., 1996; Bi and Poo, 2001; Caporale and Dan, 2008) as unsupervised weight adaptation mechanism connecting excitatory cells in the pool, a synaptic scaling mechanisms through weight normalization, and an intrinsic plasticity mechanism for firing threshold adaptation. Our approach makes use of similar mechanism in the learning procedure. Here, we are concerned with networks of gradual activation dynamics, which motivates utilizing standard Hebbian correlation learning instead of the STDP rule. Weight normalization occurs implicitly in our adaptation mechanisms by utilizing modified Hebbian learning. In particular, as discussed in Section 2.3, the bottom-up learning of receptive field weights for individual category nodes approaches a weight energy (Equations 12 and 13). The intrinsic plasticity in our scheme is accomplished through the normalization activations, or firing rates, by the pool of cells in a neighborhood defined in the space-feature domain (compare Equations 5 and 6, Brosch and Neumann, 2014a). The model of Rolfe and LeCun (2013) stresses the importance of acquisition of representations of categories and subcategories, like in our model. Their network realizes properties of deep networks establishing sparse representations of subcategories, like auto-encoder networks using binary state neuronal elements (Hinton and Salakhutdinov, 2006), and recurrently combine (hidden) representations and their predictions (Hinton et al., 2006) (see Hinton, 2007 for a review). Synaptic scaling (see a recent review in Tetzlaff et al., 2012) is addressed here from the perspective of how the receptive and projective fields learn a particular target activity distribution. In the architecture proposed by Rolfe and LeCun (2013) two types of units emerge that define parts and categories. The time course of the serial learning mechanism suggests that the network first establishes component representations mainly driven by the input. Later and with a slow learning efficacy, categories emerge that combine those units that belong to the category (while those they do not belong to are inhibited). Our proposed network architecture shares the idea of building hierarchical object representations. The acquisition of categories and subcategory, or part, representations operates oppositely: Categories are established as new representations recruiting free capacities from the long-term memory node reservoir in model layer 3 when the current input is significantly dissimilar in comparison to already existing categories. The deviations from a larger category then lead to learning subcategories and these are linked to their category representation by the temporal signature of the activation. Thus, the proposed model may start with only coarse-grained category knowledge, which is subsequently refined when more detailed information is available during the course of interacting with the environment.

While in these approaches the feedback connections serve to incorporate activations over time, feedback in ART architectures is intended to solve the stability-plasticity dilemma. The latter summarizes the necessity that an adaptive system needs to acquire or adapt to new evidence (or knowledge) and, at the same time, to keep those previously acquired representations stable (to prevent catastrophic forgetting). Our proposal differs from these previous model developments in several respects. In our architecture we build upon an abstract though biophysically plausible model of processing in cortical columns. The interaction between signal activations in bottom-up and top-down sweeps is based on modulatory feedback that enhances those sensory signal activation patterns which match the top-down template of activation that is re-entered at earlier stages of processing along the hierarchy. Thus, instead of a similarity calculation between signal patterns, a biologically plausible gain adjustment is assumed (Sherman and Guillery, 1998). The modulation signal we use for the amplification of the input signals is calculated by the difference between the current input signal and the top-down expectation pattern. This effectively combines the key mechanisms underlying the two current main theories of the role of feedback in cortex: top-down modulation and biased competition is assumed for the enhancement of the input gain. Here, the modulation strength is controlled by the difference between bottom-up and top-down signal, or the residual between these two activation patterns. Steering the amount of weight adaptation by the difference between signal and expectation template incorporates the flavor of predictive coding approaches (Rao and Ballard, 1999; Rauss et al., 2011; Bastos et al., 2012). The logic behind this strategy is that the relative enhancement is reduced monotonically the more the top-down prediction signal approaches the bottom-up signal. As a consequence, the update of the weights will more quickly converge since both, feedforward and feedback, signal remain approximately constant and the weighting pattern approaches the prediction template. Consequently, no external reset mechanism is required that explicitly detects a mismatch discrepancies by a threshold vigilance parameter, as in ART models. In our proposal, the feedback modulatory dynamics and the learning mechanisms automatically tune the average matching activation of the responding category and also select the category or feature representation. Furthermore, and potentially of even more interest is the automatic establishment of categorical representations that capture the average of the input patterns that can drive the corresponding nodes in the columnar architecture. At the same time, subcategory representations are established that represent the significant differences in the detailed feature configurations that differ from the average case. This has been demonstrated in example cases (Section 3) in which, for example, faces are distinguished from non-faces at the categorical level. Smiling facial appearances or faces where the eyes are closed are then also automatically assigned to the average category by learning. However, to distinguish the appearance differences new subcategories are automatically established and learned. This selectivity is realized by two core mechanisms. First, the realization of an associative memory through the combination of an instar with an outstar learning scheme (see Carpenter, 1989), which allows the assignment of a given input to the currently best matching internal representation, as well as the corresponding feedback pattern. Second, the modulatory amplification of the differences between an input signal and the feedback pattern associated with the best matching internal representation of the input. If the amplification after the feedback sweep is effective enough, the correlation between the modulated input and an empty category cell will be higher than to the category representation the input was assigned to in the feedforward sweep. Thus, learning will be triggered for the so far unused category cell and a new subcategory will be built.

The computational mechanisms of activation and weight dynamics support principles that have been predicted to minimize the computational efforts of visual systems to successfully deal with the complexity problem of perception (Tsotsos, 1988, 2005). The hierarchical organization of representations in model areas, the receptive field properties of model columns, the hierarchical pooling of spatially separated input representations, and the top-down feedback together with unsupervised learning are structural principles that enable the visual system to successfully cope with complex input stimuli that are behaviorally relevant. The presented model is able to build the underlying distributed representations at low, intermediate, and higher levels in the cortical hierarchy by means of key cortical principles.

### 4.3. Feedback—modulators and predictors

The hierarchical model architecture proposed here is composed of multiple model areas each of which is represented by a three-stage columnar cascade model. The cascade consists of input filtering, activity modulation of filter outputs by re-entrant signals, and competitive center-surround interaction of target cells against a pool of cells. The latter stage yields an activity normalization for generating net output responses. Together with the gain enhancement generated by input modulation via re-entrant signals the network interactions achieve a biased competition response characteristics (Desimone, 1998; Reynolds and Heeger, 2009; Carandini and Heeger, 2012). The proposed architecture can be interpreted as an abstracted compartment representation of the layered architecture of cortical areas (Self et al., 2012). The interplay between the normalization of activities and the selective enhancement of activities via feedback establishes the dynamics of cortical processing. Activity normalization at the output stage is computed by a mechanism of shunting inhibition, like the non-linear divisive mechanisms proposed in Carandini and Heeger (1994); Carandini et al. (1999); Kouh and Poggio (2008); Carandini and Heeger (2012) (see Brosch and Neumann, 2014a for a formal analysis of the computational properties). Feedback signals generated at higher-level cortical stages or parallel processing pathways provide context information that is re-entered at the current stage of the processing hierarchy (Grossberg, 1980; Edelman, 1993). While the presence of feedback connections is a well-established principle of cortical signal processing and integration, the exact role of how such feedback signals are re-entered at the earlier stages is a controversial topic of ongoing investigation. We adopt here two principles from the two major frameworks of the functionality of feedback, namely modulatory feedback to bias subsequent competitive mechanisms and predictive coding.

How feedback signals interact and combine with signals delivered in the driving feedforward stream is yet unresolved. Two major conceptual ideas have been developed, each receiving support by experimental evidence (Markov and Kennedy, 2013). In a nutshell, *biased competition* suggests that signals in the feedforward pathway are enhanced by top-down templates (represented by activity distributions) such that they receive a competitive advantage in subsequent mutually competitive processes. As a result, feature responses that receive feedback have a higher gain which, in turn, leads to stronger suppression of activities that were not enhanced (Girard and Bullier, 1989; Desimone, 1998; Roelfsema et al., 2002; Reynolds and Heeger, 2009). In *predictive coding* the goal of computation is to reduce the residual error between the feedforward signal and the (top-down) templates generated at a stage that generates an expectation about the most compatible input. This idea is based upon predictor-corrector mechanisms in optimization (Ullman, 1995; Rao and Ballard, 1999; Bastos et al., 2012). As a consequence the state trajectory of such systems and their activations are different: While in biased competition the activations of the representations that match the predictions will increase, they will decrease in the predictive coding framework. Interestingly, Spratling (2008) has shown that these two approaches are functionally equivalent when the feedback in the biased competition is additive. Here, we utilize multiplicative feedback based on the linking mechanism suggested by Eckhorn et al. (1990); Eckhorn (1999) to account for activity synchronization in networks of spiking neurons and further evidence that signal amplifications occur at the level of cortical pyramidal cells (Larkum, 2013) (see a model description in Brosch and Neumann, 2014b that accounts for these findings). An influential paper by Crick and Koch (1998) provided strong support for modulatory top-down connections based on theoretical grounds. In the model framework proposed here we adopt the framework of modulatory feedback (thus, biased competition). The feedback signals represent context-sensitive templates and are gated by feedforward driving input signals. In such a modulating feedback driven gain control mechanism spatial detail is generated by feature-driven low-level processes and representations and subsequently associated with coarse-grained context information which is provided by intermediate and higher-levels of cortical computation (Lamme and Roelfsema, 2000; Roelfsema et al., 2002; Roelfsema, 2006). In order to control the weight adaptation for learning, the strength of feedback is calculated by the difference between the feedforward signal and the predictive template that is delivered along the top-down connections. Such a difference represents the residual between the two counter stream representations (Ullman, 1995). In a nutshell, the idea is that the amount of feedback is regulated by the deviation between the two convergent streams (like in predictive coding). The re-entrant combination is, however, based on multiplicative gain enhancement. The strength of the excitatory feedback will vanish when the input is perfectly predicted by the top-down template. In that case, the feedforward signal representation will not be further enhanced. In Bastos et al. (2012) the cortical circuits are present in different compartments of a cortical area (compare Self et al., 2013 for a discussion of the possible roles of input layer and superficial and deep layer compartments in cortical area V1). Our suggested mechanism can be realized assuming subtractive interaction between driving feedforward cells and feedback signals, potentially in the superficial layer compartment. The resulting residual activations can then activate cells in columns via the apical dendrites of pyramidal cells (located either in the superficial or deep layer compartments; Larkum et al., 2004). In Brosch and Neumann (2014b) a firing-rate model of pyramidal cell interaction has been developed that explains such interactions at the level of the columnar architecture adopted here. All these feedforward and feedback interactions combine with learning mechanisms for the feedforward and the feedback connections. The equations supposed to define the weight changes lead to stable convergent weight changes. In the feedforward connection pattern the fan-in, or receptive field, weights to a unit approach a defined weight energy, or length, of the connection coefficients. This is desirable since after a representation accomplished in the weights has been settled, the activation level is not biased by the weights but is determined by the signal input and its changed gain through feedback interaction. In the feedback connection pattern the fan-out, or projective field, weights from a unit approach the (average) activity the representation is tuned to. Thus, the expected input is represented which can be activated as top-down template to instantiate the expected input signal or feature configuration. This leads to resonances in cases where the top-down expectation is retrieved from already established knowledge. In cases of mismatches new feature/category representations can be automatically recruited to establish new knowledge in the learning cortical architecture.

### 4.4. Model limitations and extensions

The proposed model architecture emphasized the computational role of feedforward and feedback mechanisms in order to generate interactive states, or resonances, in a hierarchically organized model system. The re-entrant feedback is assumed to be modulatory such that bottom-up feedforward signals gate the recurrent feedback activations. The interactive processing is combined with a learning mechanism that allows to adjust connection weights along the feedforward as well as the feedback pathways. We have demonstrated the general functionality by using simple shapes that are kept under full control during the design process. Also we employed only a pair of interacting cortical model areas, each composed as a sheet of columnar units with lateral interactions. In addition, a separate input layer that represents the stimulus was incorporated. The proposed model architecture may be investigated along several lines of questions.

In its current form, the proposed model architecture separately evaluates the activities of layer 3 category and subcategory cells before and after the modulation of the residual feedback on the input signal. This results in an activity pattern in which only an overall category cell *or* a subcategory cell can be active at a time. It would be interesting to integrate an additional mechanism which prevents such fluctuations and keeps both the overall category and the subcategory cell active in parallel.

Deep hierarchies have been proposed to accomplish the build-up of rich composite feature representations at different stages of hierarchically organized networks for solving detection and recognition tasks (LeCun et al., 1998; Hinton, 2007; Bengio, 2009). A natural extension of the simplified architecture studied in this paper is to add further model cortical areas and train the feedforward and feedback connection weights at each level. We expect that such an extended architecture allows the construction of multi-level representations of pattern compositions over several stages in a hierarchy. Such an approach should provide the generic structure to automatically build representations of fragments of input stimuli in which recognition is combined with segmenting inputs using the learned top-down templates (Ullman et al., 2002; Ullman, 2007).

The proposed scheme currently utilizes simple input patterns to build categories and associated subcategories to make explicit the variations that deviate from the average category representations. It would be interesting to study the responses for more realistic shape patterns presented as gray level inputs that provide the input to the network architecture. Also in this case, it would be interesting to study the multi-level steps necessary for the proposed model cortical architecture to accomplish the category learning under even more realistic input representations. In a technical instance of processing Borenstein and Ullman (2008) proposed an image segmentation scheme based on bottom-up signal driven processing that is combined with top-down processing to utilize knowledge for improved segmentation. Although the focus there is mainly on the improvement of image processing, the approach might serve as an inspiration for modeling as well. We suggest that the potential power of the network architecture proposed in this work lies in the automatic learning of templates for feedback expectation (at low and intermediate levels of representation; Hinton, 2007) that could be evaluated in terms of their information content for visual classification tasks (Ullman et al., 2002).

## Author contributions

Conceived and designed the experiments: Georg Layher, Heiko Neumann; Performed the experiments: Georg Layher; Analyzed the data: Georg Layher, Fabian Schrodt, Martin V. Butz, Heiko Neumann; Model implementation: Georg Layher; Wrote the paper: Georg Layher, Fabian Schrodt, Martin V. Butz, Heiko Neumann.

### Conflict of interest statement

The authors declare that the research was conducted in the absence of any commercial or financial relationships that could be construed as a potential conflict of interest.

## Footnotes

^1^We make the distinction here between feature and category representation in order to emphasize the different locality of representations that are established at different layers in a hierarchical network architecture. With increasing integration sizes of cells at different levels more information from previous stages is integrated. The zones of lateral integration are more localized at earlier stages, thus, we refer to the learning of feature representations. At later stages the convergence zones may range over the full spatial input domain and, therefore, the representations already cover categories that could be shape or motion related.
